# Prognostic value of the inflammatory burden index in predicting 1-year mortality in patients undergoing cardiac resynchronization therapy for heart failure

**DOI:** 10.3389/fcvm.2026.1934817

**Published:** 2026-10-09

**Authors:** Rustem Yilmaz, Kenan Toprak, Ahmet Karagoz, Osman Can Yontar, Mustafa Yolcu, Yasar Turan, Faruk Boyaci, Okan Oguzhan Ovaz, Emre Kasim Yilmaz, Enes Furkan Ozturk, Ersoy Öz

**Affiliations:** 1 Department of Cardiology, Faculty of Medicine, Samsun University, Samsun, Türkiye; 2 Department of Cardiology, Faculty of Medicine, Harran University, Şanlıurfa, Türkiye; 3 Department of Cardiology, Faculty of Medicine, Istinye University, Istanbul, Türkiye; 4 Department of Statistics, Yildiz Technical University, Istanbul, Türkiye

**Keywords:** cardiac resynchronization therapy, c-reactive protein, heart failure, inflammation, inflammatory burden index, mortality, prognostic marker

## Abstract

**Background/objectives:**

Heart failure (HF) is one of the leading causes of death and impairs quality of life worldwide. Systemic inflammation has been increasingly recognized as a major contributor to adverse outcomes in patients with heart failure. The Inflammatory Burden Index (IBI), a composite marker integrating C-reactive protein (CRP), neutrophils, and lymphocytes, may offer improved prognostic value over traditional inflammatory markers. This study aimed to evaluate the prognostic significance of IBI in predicting one-year mortality among patients undergoing Cardiac Resynchronization Therapy (CRT).

**Methods:**

This retrospective observational study included 525 heart failure patients who received CRT between January 2020 and January 2024. Patients were grouped based on 1-year survival status. Continuous variables were compared using Student's t-test or Mann–Whitney U test based on normality assessment, and presented as mean ± SD or median [interquartile range, IQR]. To handle right-skewness and ensure linearity in the logit, inflammatory markers were logarithmically transformed [Log(IBI) and Log(CRP)]. Multivariable logistic regression models were constructed using a standardized baseline clinical model. Incremental prognostic value was evaluated using Integrated Discrimination Improvement (IDI), continuous Net Reclassification Improvement (NRI), and DeLong's test.

**Results:**

Baseline CRP (median [IQR]: 20.90 [11.95–33.00] vs. 6.56 [3.37–13.80] mg/dL, *p* < 0.001) and IBI (median [IQR]: 81.15 [36.56–230.90] vs. 18.61 [7.76–44.46], *p* < 0.001) were significantly higher in non-survivors. In multivariable analysis, both Log(IBI) (OR: 1.983; 95% CI: 1.463–2.687; *p* < 0.001) and Log(CRP) (OR: 3.265; 95% CI: 2.278–4.678; *p* < 0.001) independently predicted 1-year mortality. Incorporating Log(IBI) or Log(CRP) significantly improved model discrimination over baseline clinical risk (AUC increased from 0.684 to 0.778 and 0.795, respectively; DeLong *p* < 0.001), yielding substantial IDI (0.0953 and 0.1118, *p* < 0.001) and continuous NRI (67.1% and 80.6%, *p* < 0.001) gains. Head-to-head comparison showed comparable discriminative performance between Log(IBI) and Log(CRP) models (DeLong *p* = 0.307).

**Conclusions:**

Both Log(IBI) and Log(CRP) are independent predictors of 1-year mortality in HF patients undergoing CRT, offering comparable incremental prognostic utility over standard clinical risk factors. Incorporating these inflammatory biomarkers into routine risk stratification may refine the identification of high-risk candidates.

## 1 Introduction

Heart failure (HF) is one of the leading causes of death and impairs quality of life worldwide. As the average life expectancy increases, the prevalence of heart failure is also increasing in both developed and developing countries. The most common causes of heart failure are chronic ischemic heart disease and hypertension (1). Overall healthcare costs for patients with chronic heart failure are increasing, and deaths occur despite optimal medical and cardiac resynchronization therapy. Prognostic assessment is becoming increasingly important in the management of these patients. The impact of systemic inflammation on heart failure prognosis has long been recognized in many studies (2, 3). Recent studies have reported that albumin, C-reactive protein (CRP), and lymphocytes predict prognosis in patients with chronic heart failure (4–7).

Previous studies have found that systemic inflammation plays an important role in the development and progression of HF and, specifically, two-thirds of patients with HF have increased biomarkers of systemic inflammation on laboratory tests and that high inflammatory burden is associated with a significantly higher incidence of HF-related hospitalizations and deaths (8, 9). The systemic inflammatory response is especially evident in patients with acute HF. Comorbidities and acute stress factors in these patients may trigger activation of the nucleotide-binding oligomerization domain-leucine-rich repeat and pyrin domain-containing protein 3 inflammasome, leading to the release of the proinflammatory cytokines IL (interleukin)-1ß and IL-18 (10). CRP (C-reactive protein) is an inflammatory protein produced by the liver in response to the cytokine IL-6, which is activated by IL-1. For this reason, CRP is widely used as a biomarker of inflammation reflecting IL-1 activity, which has been found to have direct cardio-depressant effects in cardiac cells and animal models and increased activity in patients with HF (11, 12). Even minimally elevated CRP measured by high-sensitivity tests [hs-CRP (high-sensitivity CRP)] has been shown to be associated with a worse prognosis in patients hospitalized with HF (13).

Cardiac resynchronization therapy (CRT) is an effective interventional treatment for patients with ischemic or non-ischemic dilated cardiomyopathy, QRS width greater than 150 milliseconds (ms), left bundle branch block pattern on ECG, NYHA III and IV capacity despite optimal medical therapy, and ejection fraction less than 35%. CRT improves exercise capacity and reduces heart failure hospitalization and mortality (14). However, not all patients experience the same improvement from CRT (15). One study reported that female gender, non-ischemic cardiomyopathy, left bundle branch block (LBBB), longer QRS duration, and sinus rhythm were associated with a positive response to CRT (16). However, these patients may not benefit from CRT either, and there are still limited data to determine this. Approximately one-third of patients do not respond to CRT. Several factors may account for this, including lack of appropriate CRT optimization, anemia, and inadequate coronary sinus branches for left ventricular stimulation (15, 17). Systemic inflammatory mediators and immune cells (lymphocytes, neutrophils, and platelets) play an important role in the pathogenesis of myocardial dysfunction (18). However, the pathophysiologic basis of the interaction between leukocyte subsets, inflammatory markers, and heart failure is multifactorial (19). Previous studies have found that the neutrophil-to-lymphocyte ratio (NLR) is effective in predicting response to CRT (20–22). A recent study found that the systemic immune-inflammation index is effective in predicting non-response to CRT (23). As a result, failure to respond to CRT may increase the frequency of hospitalization and death.

Inflammatory burden index (IBI) has been proposed as a new inflammatory biomarker to assess the degree of local or systemic inflammatory reactions, incorporating three inflammatory markers: CRP, neutrophils, and lymphocytes (24). A study found that IBI better indicated the degree of systemic inflammation compared to CRP or NLR biomarkers alone. Several studies have also shown that IBI has a better predictive value for the prognosis of different diseases (24, 25). A recent study found that elevated IBI was associated with an increased risk of all-cause mortality in the general population over the age of 45 (26). Based on these findings, it has been hypothesized that IBI may provide additional prognostic value over individual inflammatory markers in patients undergoing CRT for heart failure. The aim of our study is to investigate the prognostic value of the inflammatory burden index in predicting one-year mortality in patients undergoing cardiac resynchronization therapy for HF.

## 2 Materials and methods

### 2.1 Study design and dataset

This investigation adopts a retrospective observational design, including a cohort of patients undergoing cardiac resynchronization therapy for heart failure at our medical institution between January 2020 and January 2024. The cohort was subsequently dichotomized into two subsets based on 1-year mortality status following CRT pacemaker implantation, comprising a total of 525 individuals (Group 0: Survivor and Group 1: Non-survivor). Vital status at exactly 365 days post-implantation was verified for all participants via the Ministry of Health National Death Registry System, achieving 100% complete follow-up with zero loss to follow-up or uninformative censoring prior to 1 year. This study was conducted in accordance with the Declaration of Helsinki and approved by the Clinical Research Ethics Committee of Samsun University (Date: 07.02.2025; Protocol number: 2024/18/2).

### 2.2 Inclusion and exclusion criteria

Patients who underwent CRT in our clinic due to heart failure, who had ischemic or non-ischemic dilated cardiomyopathy, QRS width greater than 150 ms, left bundle branch block pattern on ECG, NYHA III and IV capacity despite optimal medical treatment, and ejection fraction less than 35% were included in this study.

The exclusion criteria are given below:

(a) Age <18 years and >80 years,
(b) A hemoglobin value of less than 8 g/dL,
(c) Acute infections,
(d) Severe renal impairment [estimated glomerular filtration rate (eGFR) < 15 mL/min/1.73 m^2^],
(e) A history of cancer detected within the previous year,
(f) Severe liver impairment,
(g) Presence of missing data about the patient.

### 2.3 Data collection

Data pertaining to laboratory results, echocardiography findings, demographic variables, and comorbidities were extracted from electronic medical records. The hematological and biochemical parameters including urea (mg/dL), creatinine (mg/dL), albumin (g/dL), C-reactive protein (CRP, reported in mg/dL; reference range <0.5 mg/dL), aspartate transaminase (AST), alkaline phosphatase (ALT) and hemoglobin (HGB) levels as well as platelet (PLT), white blood cell (WBC), neutrophil (NEU), lymphocyte (LY), neutrophil-lymphocyte ratio (NLR), and monocytes (MO) counts were ascertained from venous blood samples obtained during the initial cardiology service admission.

Venous blood samples for routine laboratory testing, including C-reactive protein and complete blood counts, were drawn from all patients during the initial hospital admission prior to the CRT implantation procedure (within 24–48 h before device placement). All patients were clinically stable at the time of blood sampling, and individuals with active acute infections, active systemic inflammatory/autoimmune disorders, or malignancy were strictly excluded per the exclusion criteria.

The inflammatory burden index was computed using a standardized formula: C-reactive protein  ×  (Neutrophils/Lymphocytes ratio) serving as a key variable in the study’s predictive model.

### 2.4 Statistical analysis

Patients were categorized into two groups based on their 1-year survival status following CRT: Survivor and Non-Survivor. Categorical variables were compared using the Chi-square test. Continuous variables were evaluated for normality using the Kolmogorov–Smirnov test and visual inspection of histograms. Normally distributed continuous variables are presented as mean ± standard deviation (SD) and were compared using the Independent Samples t-test. Skewed continuous variables (including WBC, neutrophils, lymphocytes, monocytes, CRP, serum creatinine, NLR, and IBI) are expressed as median [interquartile range, IQR: 25th–75th percentiles] and were compared using the Mann–Whitney U test. Categorical variables are presented as frequencies and percentages, and group differences were evaluated using the Pearson Chi-square test or Fisher's exact test, as appropriate. To identify independent predictors of 1-year mortality and avoid multicollinearity arising from mathematical dependencies among composite indices and their parent components (e.g., IBI = CRP   ×   NLR), separate multivariable logistic regression models were constructed. Due to marked right-skewness, continuous inflammatory biomarkers were logarithmically transformed [Log(IBI), Log(CRP), and Log(NLR)] prior to multivariable modeling to ensure linearity in the logit. A standardized Baseline Clinical Model was initially established using established risk factors (age, gender, hemoglobin, serum creatinine, ejection fraction, coronary artery disease, and diabetes mellitus). Odds ratios (ORs), 95% confidence intervals (CIs), and *p*-values were reported for each model. Multicollinearity was systematically assessed using the Variance Inflation Factor (VIF < 2.0 considered acceptable). Model performances were evaluated using receiver operating characteristic (ROC) curve analysis and Area Under the Curve (AUC). The incremental prognostic value of adding Log(IBI) or Log(CRP) to the baseline model was evaluated using pairwise AUC comparisons (DeLong test), Integrated Discrimination Improvement (IDI), and continuous Net Reclassification Improvement (NRI). A two-tailed *p*-value < 0.05 was considered statistically significant in all analyses. Internal validation and assessment of model optimism were performed using 1,000 bootstrap resamples. Model calibration was evaluated using the Hosmer-Lemeshow goodness-of-fit test, and linearity in the logit for continuous variables was formally tested using Box-Tidwell interaction terms (*X.*ln*(X)*).

## 3 Results

### 3.1 Baseline characteristics

The baseline demographic, clinical, laboratory, and echocardiographic parameters of the survivor and non-survivor groups are summarized in Table 1. Continuous variables are expressed as mean ± standard deviation or median [interquartile range] based on their normality distribution, while categorical variables are reported as counts and percentages.

**Table 1:** Baseline demographic, clinical, laboratory, and echocardiographic characteristics of the study cohort according to 1-year mortality status.

Variable	Survivor (*N* = 442)	Non-survivor (*N* = 83)	*p*-value	Statistical test
Demographic and Clinical Characteristics				
AGE year, mean ± SD	67.1 ± 9.2	69.1 ± 7.4	0.090	Independent Samples t
GENDER Male, n(%)	307 (69.5)	61 (73.5)	0.544	Chi-square
DM Yes, n(%)	213 (48.2)	38 (45.8)	0.678	Chi-square
HT Yes, n(%)	405 (91.6)	76 (91.6)	1.000	Chi-square
CAD Yes, n(%)	292 (66.1)	53 (63.9)	0.793	Chi-square
Hematological-Biochemical- Echocardiographic				
WBC (10^9^/L), median [IQR]	8.14[6.85−9.91]	9.22[7.19−12.62]	<0.001***	Mann–Whitney U
HGB (g/dL), mean ± SD	12.7 ± 2.0	11.7 ± 2.1	<0.001***	Independent Samples t
PLT (10^9^/L), mean ± SD	225.7 ± 73.1	225.3 ± 82.9	0.652	Independent Samples t
NEU (10^9^/L), median [IQR]	5.30[4.20−6.73]	6.69[4.66−9.39]	<0.001***	Mann–Whitney U
LY (10^9^/L), median [IQR]	1.80[1.30−2.29]	1.40[1.02−2.05]	0.002**	Mann–Whitney U
MO (10^9^/L), median [IQR]	0.66[0.51−0.85]	0.76[0.50−1.04]	0.019*	Mann–Whitney U
CRP (mg/dL), median [IQR]	6.56[3.37−13.80]	20.90[11.95−33.00]	<0.001***	Mann–Whitney U
CREATININ (mg/dL), median [IQR]	1.11[0.90−1.40]	1.30[0.95−2.02]	0.001***	Mann–Whitney U
EF %, mean ± SD	28.6 ± 5.0	27.2 ± 4.6	0.015*	Independent Samples t
NLR, median [IQR]	2.90[2.05−4.39]	4.33[2.56−7.87]	<0.001***	Mann–Whitney U
IBI, median [IQR]	18.61[7.76−44.46]	81.15[36.56−230.90]	<0.001***	Mann–Whitney U

Values are presented as mean ± standard deviation for normally distributed continuous variables, median [interquartile range, 25th–75th percentiles] for skewed continuous variables, or frequency (percentage) for categorical variables. Categorical variables were compared using the Pearson Chi-square test. Continuous variables were compared using the Independent Samples t-test or the Mann–Whitney U test based on normality assessment (Kolmogorov–Smirnov test). DM, diabetes mellitus; HT, hypertension; CAD, coronary artery disease; WBC, white blood cell; HGB, hemoglobin; PLT, platelet; NEU, neutrophil; LY, lymphocyte; MO, monocyte; CRP, C-reactive protein; EF, ejection fraction; NLR, neutrophil-to-lymphocyte ratio; IBI, inflammatory burden index; SD, standard deviation. *Difference is significant at the 95% confidence level (two-tailed). **Difference is significant at the 99% confidence level (two-tailed). ***Difference is significant at the 99.9% confidence level (two-tailed).

Table 1 summarizes the baseline clinical, laboratory, and echocardiographic characteristics of the study population according to 1-year mortality status. No statistically significant differences were observed between survivors and non-survivors regarding demographic and clinical comorbidities, including age (*p* = 0.090), male gender (69.5% vs. 73.5%, *p* = 0.544), diabetes mellitus (48.2% vs. 45.8%, *p* = 0.678), hypertension (91.6% vs. 91.6%, *p* = 1.000), and coronary artery disease (66.1% vs. 63.9%, *p* = 0.793).

In addition to categorical variables, several hematological, biochemical, and echocardiographic parameters exhibited statistically significant differences between the Survivor and Non-Survivor groups. Age was slightly higher in the non-survivor group; however, the difference was not statistically significant (*p* = 0.090). Similarly, platelet (PLT) levels did not show a significant difference between the two groups (*p* = 0.652). Compared with survivors, non-survivors demonstrated significantly elevated baseline levels of systemic inflammatory and hematological markers, including C-reactive protein (median [IQR]: 20.90 [11.95–33.00] vs. 6.56 [3.37–13.80] mg/dL, *p* < 0.001), Inflammatory Burden Index (median [IQR]: 81.15 [36.56–230.90] vs. 18.61 [7.76–44.46], *p* < 0.001), neutrophil-to-lymphocyte ratio (median [IQR]: 4.33 [2.56–7.87] vs. 2.90 [2.05–4.39], *p* < 0.001), white blood cell count (*p* < 0.001), neutrophil count (*p* < 0.001), monocyte count (*p* = 0.019), and serum creatinine (*p* = 0.001). Conversely, lymphocyte count (median [IQR]: 1.40 [1.02–2.05] vs. 1.80 [1.30–2.29] × 10⁹/L, *p* = 0.002), hemoglobin levels (11.7 ± 2.1 vs. 12.7 ± 2.0 g/dL, *p* < 0.001), and left ventricular ejection fraction (27.2 ± 4.6% vs. 28.6 ± 5.0%, *p* = 0.015) were significantly lower in the non-survivor group.

### 3.2 Multivariable logistic regression models

To identify independent predictors of 1-year mortality among patients undergoing CRT and to evaluate the prognostic value of systemic inflammatory markers without introducing multicollinearity, multivariable logistic regression analyses were conducted on a standardized Baseline Clinical Model. This baseline model incorporated established clinical, laboratory, and echocardiographic parameters, including age, gender, hemoglobin, serum creatinine, ejection fraction, coronary artery disease, and diabetes mellitus (Baseline Model AUC = 0.684). To address marked right-skewness and satisfy the assumption of linearity in the logit, continuous inflammatory biomarkers were logarithmically transformed [Log(IBI), Log(CRP), and Log(NLR)] prior to multivariable modeling. Linearity in the logit was formally confirmed using Box-Tidwell interaction tests, demonstrating no violation of linearity for Log(CRP) (*p* = 0.245), Log(IBI) (*p* = 0.684), or Log(NLR) (*p* = 0.056). Because IBI is mathematically derived from CRP and NLR (IBI = CRP × NLR), composite indices and their constituent parent components were evaluated in separate, non-overlapping regression models to preserve model stability and interpretability. In Model A, incorporating Log(IBI) alongside baseline covariates, higher Log(IBI) was confirmed as a robust independent predictor of 1-year mortality (OR: 1.983; 95% CI: 1.463–2.687; *p* < 0.001). Lower hemoglobin levels (OR: 0.840; 95% CI: 0.724–0.976; *p* = 0.023) and reduced ejection fraction (OR: 0.943; 95% CI: 0.894–0.994; *p* = 0.029) also maintained statistically significant independent associations with 1-year mortality. In Model B, designed to assess C-reactive protein, elevated Log(CRP) independently predicted 1-year mortality (OR: 3.265; 95% CI: 2.278–4.678; *p* < 0.001). Reduced ejection fraction remained significant (OR: 0.939; 95% CI: 0.889–0.992; *p* = 0.025). Multicollinearity diagnostics confirmed acceptable variance inflation factors across both finalized models, with all VIF values remaining below 1.35. These multivariable logistic regression results are presented in Table 2. Internal bootstrap validation (1,000 resamples) demonstrated high stability and minimal optimism, with bias-corrected AUCs of 0.771 for Model A [Log(IBI)] and 0.788 for Model B [Log(CRP)]. Both finalized models showed no evidence of significant lack of fit based on the Hosmer-Lemeshow goodness-of-fit test (Model A: *χ*^2^ = 6.12, *p* = 0.633; Model B: *χ*^2^ = 7.84, *p* = 0.449).

**Table 2:** Multivariable logistic regression analysis for predicting 1-year mortality incorporating logarithmic transformations of IBI and CRP.

Model/Variable	B	SE	Wald	*p*-value	OR (Exp(B))	95% CI for OR	VIF
Model A: Baseline + Log(IBI)							
AGE, years	23	17	1.742	187	1.023	0.989–1.058	1.12
GENDER (Male)	12	316	1	970	1.012	0.545–1.881	1.19
HGB, g/dL	−0.174	76	5.182	0.023*	840	0.724–0.976	1.34
CREATININE, mg/dL	182	158	1.325	250	1.200	0.880–1.636	1.15
EF, %	−0.059	27	4.761	0.029*	943	0.894–0.994	1.06
CAD	18	278	4	948	1.018	0.591–1.755	1.05
DM	165	268	381	537	1.180	0.698–1.996	1.02
Log(IBI)	685	156	19.124	<0.001**	1.983	1.463–2.687	1.19
Model B: Baseline + Log(CRP)							
AGE, years	28	18	2.482	115	1.028	0.993–1.065	1.12
GENDER (Male)	141	328	185	667	1.151	0.606–2.189	1.18
HGB, g/dL	−0.118	78	2.261	133	889	0.762–1.037	1.30
CREATININE, mg/dL	171	161	1.134	287	1.187	0.865–1.629	1.13
EF, %	−0.063	28	5.012	0.025*	939	0.889–0.992	1.04
CAD	−0.158	291	295	587	854	0.483–1.510	1.05
DM	128	278	211	646	1.136	0.698–1.959	1.02
Log(CRP)	1.183	184	41.341	<0.001**	3.265	2.278–4.678	1.09

OR, ddds ratio; CI, confidence interval; VIF, variance inflation factor; DM, diabetes mellitus; CAD, coronary artery disease; HGB, hemoglobin; EF, ejection fraction; IBI, inflammatory burden index; CRP, C-reactive protein. *Significant at 95% confidence level. **Significant at 99.9% confidence level.

### 3.3 Assessment of incremental prognostic value and model discrimination

To determine whether Log(IBI) and Log(CRP) provide incremental prognostic value over standard risk factors, discriminative performance, reclassification improvement, and integrated discrimination metrics were evaluated against the Baseline Clinical Model.

As detailed in Table 3, the Baseline Clinical Model achieved a moderate discriminative capability (AUC = 0.684; 95% CI: 0.621–0.747). Incorporating Log(IBI) significantly enhanced overall model discrimination, increasing the AUC to 0.778 (95% CI: 0.722–0.834; DeLong test vs. Baseline *p* = 0.0006). This addition yielded a substantial Integrated Discrimination Improvement (IDI) of 0.0953 (*p* < 0.001) and a continuous Net Reclassification Improvement (NRI) of 67.1% (*p* < 0.001), confirming significant reclassification benefit over baseline clinical parameters.

**Table 3:** Assessment of incremental prognostic value and discriminative performance over baseline clinical risk.

Model	AUC (95% CI)	*Δ* AUC vs. Baseline	DeLong *p*-value vs. Baseline	IDI (95% CI)	Continuous NRI (95% CI)
Baseline Clinical Model	0.684 (0.621–0.747)	Reference	Reference	Reference	Reference
Baseline + Log(IBI)	0.778 (0.722–0.834)	+0.094	0.0006***	0.0953 (*p* < 0.001)	67.1% (*p* < 0.001)
Baseline + Log(CRP)	0.795 (0.741–0.849)	+0.111	0.0001***	0.1118 (*p* < 0.001)	80.6% (*p* < 0.001)
Head-to-Head Comparison:Log(IBI) Model vs. Log(CRP) Model	−	−0.017	0.3071 (NS)	−	−

AUC, area under the curve; IDI, integrated discrimination improvement; NRI, net reclassification improvement; NS, not significant (*p* > 0.05). ***Significant at 99.9% confidence level.

Similarly, adding Log(CRP) to the baseline model led to a significant gain in discriminative ability, elevating the AUC to 0.795 (95% CI: 0.741–0.849; DeLong test vs. Baseline *p* = 0.0001), accompanied by an IDI of 0.1118 (*p* < 0.001) and a continuous NRI of 80.6% (*p* < 0.001).

Direct pairwise head-to-head comparison between the multivariable model with Log(IBI) (Model A) and the model with Log(CRP) (Model B) demonstrated no statistically significant difference in discriminative performance (AUC difference: −0.017; DeLong test *p* = 0.3071). These findings indicate that both inflammatory biomarkers provide meaningful, comparable incremental prognostic utility over baseline clinical parameters in predicting 1-year mortality following CRT. The corresponding ROC curves for the Baseline Clinical Model, Baseline + Log(IBI), and Baseline + Log(CRP) models are illustrated in Figure 1.

**Figure 1:**
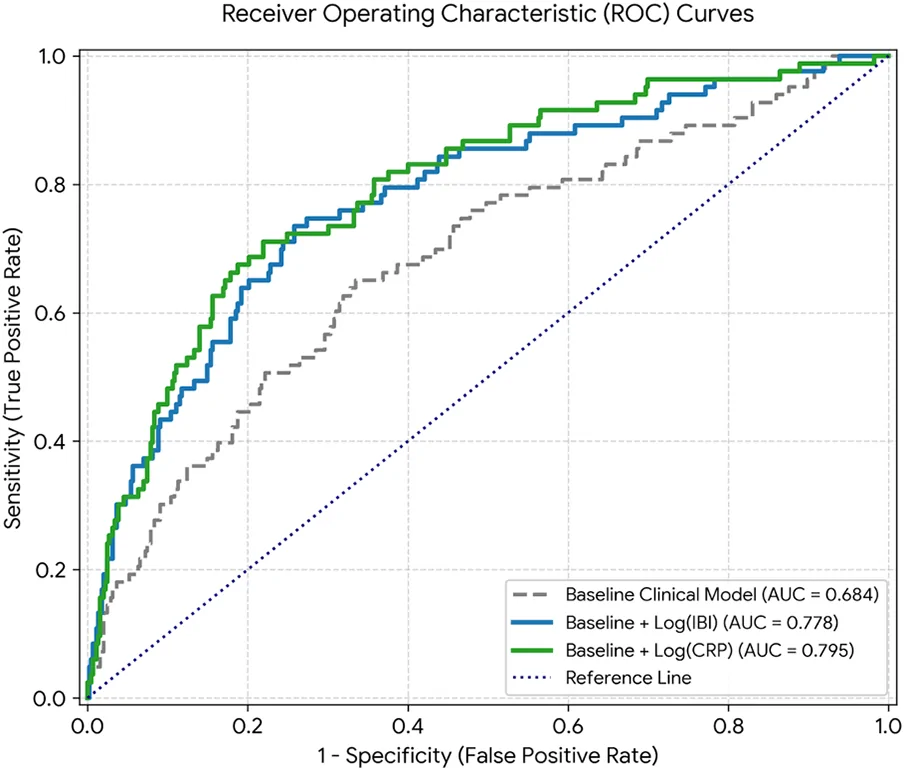
Receiver operating characteristic (ROC) curves comparing the discriminative performance of the baseline clinical model (AUC = 0.684), baseline + log(IBI) model (AUC = 0.778), and baseline + log(CRP) model (AUC = 0.795) for predicting 1-year mortality following CRT.

## 4 Discussion

CRT has revolutionized the management of advanced heart failure by improving symptoms, enhancing left ventricular systolic function, and reducing mortality in selected patient populations (14). Nevertheless, outcomes remain highly heterogeneous, with a substantial proportion of patients failing to derive significant benefit from CRT despite appropriate implantation (15). This variability underscores the necessity for improved risk stratification strategies that integrate conventional clinical predictors with novel biomarkers reflecting underlying disease biology. In this context, systemic inflammation has garnered considerable interest as a pathophysiological determinant of heart failure progression and device-based therapy outcomes (20–23).

The role of inflammation in cardiovascular disease has been well established, with numerous studies linking chronic low-grade immune activation to myocardial remodeling, neurohormonal dysregulation, and adverse outcomes. Elevated levels of inflammatory markers such as C-reactive protein (CRP), interleukin-6, and tumor necrosis factor-α have been consistently associated with mortality and hospitalization in heart failure cohorts (4–7, 11–13). Mechanistically, inflammation contributes to extracellular matrix remodeling, endothelial dysfunction, arrhythmogenesis, and progressive ventricular dilatation, all of which are highly relevant to CRT candidacy (27). Thus, inflammatory biomarkers provide not only prognostic insights but also a potential therapeutic target in heart failure populations.

CRT response is multifactorial and influenced by baseline electrical, structural, and biological factors. Classical predictors of favorable CRT response include female sex, non-ischemic etiology, presence of LBBB with QRS duration ≥150 ms, and preserved right ventricular function (28–32). Conversely, ischemic cardiomyopathy, severe LV dilatation, absence of LBBB, right ventricular dysfunction, and suboptimal lead positioning have consistently been associated with CRT non-response (33–36). Although these predictors are essential for patient selection, they do not fully capture the biological complexity underlying CRT outcomes.

Emerging data suggest that inflammatory status may complement traditional predictors, adding another layer of risk stratification. Previous studies demonstrated that both CRP and neutrophil-to-lymphocyte ratio (NLR) are associated with CRT outcomes and long-term prognosis in heart failure cohorts (4–7). More recently, composite inflammatory indices such as the Inflammatory Burden Index (IBI), which integrates CRP, neutrophil, and lymphocyte counts, have been proposed to better reflect systemic immune dysregulation (24–26). However, their incremental prognostic value over established single biomarkers such as CRP remains uncertain. In our cohort, standard cardiovascular comorbidities including diabetes mellitus and hypertension did not significantly differ between survivors and non-survivors. Notably, hypertension was highly prevalent across the entire study population (91.6%), suggesting that traditional comorbidities alone offer limited discriminative capacity in advanced heart failure candidates undergoing CRT. Specifically, non-survivors exhibited a markedly heightened baseline systemic inflammatory tone compared to survivors, as reflected by substantially higher median CRP (20.90 vs. 6.56 mg/dL, *p* < 0.001) and IBI values (81.15 vs. 18.61, *p* < 0.001).

Our updated multivariable modeling directly compared the incremental prognostic value of Log(IBI) and Log(CRP) over a standardized baseline clinical risk model in predicting one-year mortality after CRT implantation. Following logarithmic transformations to ensure linearity in the logit, both Log(IBI) (OR: 1.983, *p* < 0.001) and Log(CRP) (OR: 3.265, *p* < 0.001) were confirmed as independent predictors of post-CRT mortality (11–13). Crucially, when evaluating model performance beyond traditional ROC curve analysis, adding either Log(IBI) or Log(CRP) to baseline clinical variables yielded significant reclassification and discrimination gains, as evidenced by substantial Integrated Discrimination Improvements (IDI = 0.0953 for IBI, IDI = 0.1118 for CRP; both *p* < 0.001) and continuous Net Reclassification Improvements (NRI = 67.1% for IBI, NRI = 80.6% for CRP; both *p* < 0.001) (4–7). Direct head-to-head comparison between the Log(IBI) and Log(CRP) multivariable models revealed no statistically significant difference in discriminative capacity (AUC 0.778 vs. 0.795, DeLong *p* = 0.3071), indicating that both systemic inflammatory indices offer comparable incremental utility over baseline clinical parameters. Interestingly, left ventricular ejection fraction, while consistently significant in all multivariate models, exhibited lower predictive strength compared to inflammatory markers. This finding highlights the evolving recognition that ejection fraction alone is insufficient to fully describe the complex interplay between structural remodeling, electrophysiological desynchrony, and systemic inflammation in determining CRT response (37). Integrative risk stratification models that incorporate both echocardiographic and inflammatory parameters may thus provide a more nuanced and clinically useful prognostic framework.

From a clinical perspective, our findings reinforce the central role of inflammation in shaping outcomes after CRT. CRP, as a widely available and inexpensive biomarker, may serve as a practical tool for refining risk stratification and identifying high-risk patients who warrant closer follow-up or adjunctive therapies. Although Log(IBI) yielded comparable discriminative capability to Log(CRP) without statistical superiority, its composite nature integrates both acute-phase humoral inflammation and cellular immune dysregulation. Whether combining non-redundant cellular inflammatory pathways with acute-phase reactants within multiparametric risk scores offers additive utility warrants further prospective investigation. Whether targeted anti-inflammatory strategies—such as interleukin-1 or interleukin-6 modulation—could specifically benefit CRT candidates with heightened baseline inflammatory burden remains a compelling area that merits future prospective interventional investigation (38).

## 5 Limitations

Our study has several important limitations that warrant consideration. First, its retrospective, single-center design introduces inherent risks of selection bias and unmeasured confounding, which may limit the generalizability of our findings to broader, multicenter CRT populations. Second, inflammatory biomarkers were assessed exclusively at baseline prior to device implantation, without accounting for potential post-procedural dynamic fluctuations over time. Serial measurements of CRP and IBI could offer superior prognostic insights into the temporal resolution of systemic inflammation following CRT response (4–7). Third, although multivariable logistic regression adjusted for core clinical, biochemical, and echocardiographic covariates, certain specific CRT-related parameters—such as exact QRS duration/morphology sub-phenotypes (e.g., LBBB vs. non-LBBB details), baseline right ventricular systolic function, and specific neurohormonal medication dosages—were not completely recorded across the entire cohort and could not be included in the multivariable models. Future prospective, multicenter studies incorporating serial biomarker tracking and comprehensive electrophysiological registries are required to externally validate these findings and further refine inflammatory risk-stratification algorithms in CRT candidates.

## 6 Conclusions

In summary, the current study adds to the growing body of evidence linking systemic inflammation with adverse outcomes in heart failure and CRT populations. Consistent with prior work, we confirm that CRP is a powerful and independent predictor of mortality. Importantly, we extend existing knowledge by systematically comparing CRP with IBI, demonstrating that both markers are independent predictors that offer comparable incremental prognostic value over standard baseline clinical risk factors. The prognostic role of inflammatory biomarkers further suggests that integrative models incorporating multiple inflammatory and structural parameters may be the most effective approach for risk stratification.

By situating our findings within the broader literature, it becomes clear that systemic inflammation is not merely a bystander but is significantly associated with adverse outcomes following CRT. Future prospective studies should validate these observations, assess serial biomarker changes over time, and explore whether combining systemic inflammatory biomarkers with complementary clinical, electrophysiological, and structural parameters within multiparametric frameworks enhances prediction of mortality and CRT non-response. Ultimately, integrating inflammatory biomarkers into personalized management algorithms could refine patient selection, guide follow-up intensity, and inform the development of novel therapeutic strategies targeting inflammation in this high-risk population.

## Data Availability

The original contributions presented in the study are included in the article/Supplementary Material, further inquiries can be directed to the corresponding author.
